# Comparison of the Characteristics of Brucella Patients Diagnosed With Blood Culture Positivity and/or Serology

**DOI:** 10.7759/cureus.43758

**Published:** 2023-08-19

**Authors:** Petek Konya, Nese Demirturk, Melahat Gürbüz, Gamze Colak

**Affiliations:** 1 Infectious Diseases, Afyonkarahisar Health Sciences University, Afyonkarahisar, TUR; 2 Clinical Microbiology, Afyonkarahisar Health Sciences University, Afyonkarahisar, TUR

**Keywords:** infection, rose bengal dye, serology, blood culture, brucellosis

## Abstract

Purpose: The aim of this study is to investigate and compare the clinical, laboratory, and treatment response characteristics of patients diagnosed with positive culture or serology. In this way, we wanted to assess the validity of serological diagnosis.

Materials and methods: The study was designed as a retrospective cross-sectional study between January 2010 and 2020. Patients with clinical and laboratory findings of acute/subacute brucellosis, patients with positive serological tests, and patients with growth of *Brucella* spp. in blood culture were included in the study. The patients were divided into three groups: Group 1 - Wright agglutination test result ≥ 1/160 and *Brucella* spp. growth in blood culture; Group 2 - Wright agglutination test result ≥ 1/160 and no growth in blood culture; and Group 3 - *Brucella* spp. growth in blood culture and negative serological test.

These three groups were retrospectively evaluated for clinical features, laboratory parameters, areas of involvement, treatment options, and treatment response.

Results: We identified 294 patients diagnosed with brucellosis. Blood cultures were obtained from all patients, and *Brucella *spp. was detected in 40 patients (13.6%). There were 35 patients in Group 1, 254 patients in Group 2, and five patients in Group 3. When examining patients with symptoms, only fever showed a difference between the groups, which was significantly higher in Group 1. Laboratory investigations of the C-reactive protein (CRP), aspartate aminotransferase (AST), and alanine aminotransferase (ALT) levels of the patients showed significant differences between the groups; these parameters were significantly higher in Group 1.

Conclusion: No significant difference was found in terms of treatment response and prognosis between patients with and without blood culture growth who were clinically compatible with acute/subacute brucellosis as diagnosed by serological methods. Therefore, serological tests are reliable methods for the diagnosis of brucellosis in cases where blood culture is inconclusive.

## Introduction

Brucellosis is a common zoonosis and is considered the most common laboratory-acquired infection worldwide. Approximately 500,000 cases of human brucellosis are reported annually. However, the actual incidence is estimated to be between 5,000,000 and 12,500,000 cases per year [1]. According to the data of the Turkish Public Health Institution, in 2017, the number of brucellosis cases in Turkey was 6,457, with a morbidity rate of 7.99 per 100,000 with the highest incidence in the Southeastern and Eastern Anatolia regions [2].

As brucellosis can affect any organ and system, the presenting symptoms of the infection are not pathognomonic; therefore, the disease can be easily confused with other medical conditions [3]. Accurate diagnosis of brucellosis in humans is therefore of critical importance not only for early and appropriate patient management but also for public health as it may reveal exposure to infected animals, consumption of contaminated food (particularly dairy products), and violation of laboratory safety practices. Routine laboratory tests for the diagnosis of brucellosis include culture, polymerase chain reaction (PCR), and serological tests based on the detection of antibodies. The most common of these is the tube agglutination test. A definitive diagnosis of brucellosis is the culture of blood, bone marrow, tissues, or retained body fluids such as joint fluid and cerebrospinal fluid. Automated blood culture systems provide faster results than conventional culture methods, often with a positive signal within a week. Because of the low sensitivity of positive blood culture, which is considered the gold standard for diagnosing brucellosis, serological tests and compatible clinical findings are commonly used in practice [4].

In this study, we aimed to determine the blood culture positivity rates in patients diagnosed serologically; to examine and compare the clinical, laboratory, and treatment response characteristics of culture-positive and culture-negative patients; and to evaluate the validity of serological diagnosis.

## Materials and methods

Approval for our study was obtained from the Ethics Committee of Afyonkarahisar Health Sciences University on June 3, 2022 (2022/320). Informed consent was obtained from all individual participants included in the study.

The study was designed as a cross-sectional retrospective study between January 2010 and 2020. Patients diagnosed with acute/subacute brucellosis based on clinical and laboratory findings, patients with positive serological tests (standard tube agglutination test [STA, Wright] ≥ 1/160), and patients with positive blood cultures for *Brucella *spp. were included in this study. Exclusion criteria were age ≤ 18 and STA ˂ 1/160.

After identifying all patients, it was determined whether blood cultures were obtained from patients who received a serological diagnosis and whether positive results were identified in these patients. Subsequently, patients were divided into three groups as follows:

Group 1: Patients with Wright agglutination test results ≥ 1/160 and *Brucella *spp. growth in blood culture.

Group 2: Patients with Wright agglutination test results ≥ 1/160 and no growth in blood culture.

Group 3: Patients with negative Wright agglutination test and diagnosed with *Brucella *spp. blood culture positivity.

In our study, the clinical features, laboratory parameters, sites of involvement, treatment options, and treatment responses of the three groups were retrospectively evaluated and compared.

Three criteria were defined to evaluate patients' treatment responses: (1) cure - complete clinical improvement and normalization of non-specific laboratory tests; (2) relapse - recurrence of symptoms within three months after treatment discontinuation; and (3) non-response - no improvement in clinical and/or laboratory findings.

In routine practice, patient blood samples sent for serological testing are centrifuged at 4,000 rpm for 10 min, and the separated serum is first applied as a screening test for all patient samples using the Rose Bengal plate agglutination test (Seromed, Turkey). Rose Bengal test antigen, a standardized suspension prepared from the *Brucella abortus* S99 strain, was mixed with the patient’s serum, rotated on a rotator for four minutes, and examined for agglutination. The samples with agglutination were considered positive, otherwise negative. STA (bioMedica, Canada) test was performed on the sera of the patients with positive screening results. For the standard tube agglutination test, serial dilutions ranging from 1/10 to 1/1280 were prepared using the patient's serum with saline, and 500 µL of the *Brucella *STA test antigen was pipetted onto each tube to obtain a final dilution of 1/20-1/2560. Positive and negative controls were used in each study by the manufacturer's recommendations. The tubes were shaken to homogenize the serum and antigen mixture and incubated at 37°C for 24 hours. Agglutination observed at a titer of 1/160 and above was considered positive, and the highest titer value for agglutination was reported.

Blood cultures were incubated for five days in an automated BACT/ALERT 3D device (bioMérieux, Marcy l'Etoile, France) in routine practice during the retrospective screening period. If extended incubation was required, the bottles were incubated for up to 10 days. Blood culture bottles with a positive signal were subcultured on sheep blood agar, eosin methylene blue agar (EMB), and chocolate agar plates. Sheep blood and EMB agar plates were incubated in aerobic conditions, while chocolate agar plates were incubated in a 5%-10% CO_2_ atmosphere. After 24-48 hours of incubation, the colonies were evaluated, and gram staining and biochemical tests were performed. Gram-negative *coccobacilli*, catalase, and oxidase positive, not growing on EMB agar, forming black color due to H2S production on triple sugar iron (TSI) agar, and demonstrating urease activity on Christensen's urea agar were identified as *Brucella *spp.

Statistics

Data were evaluated using descriptive statistics (arithmetic mean, median, standard deviation, and percentage distribution). When comparing the mean between groups, suitability for normal distribution was assessed using the Kolmogorov-Smirnov and Shapiro-Wilk tests.

When comparing the means of two dependent groups, the paired sample t-test was used when parametric conditions were met, and the Wilcoxon test was used when parametric conditions were not met. When comparing the means of two independent groups, the independent samples t-test was used when parametric conditions were met, and the Mann-Whitney U test was used when parametric conditions were not met. When comparing the means of three or more independent groups, one-way analysis of variance (ANOVA) was used when parametric conditions were met, and the Kruskal-Wallis test was used when parametric conditions were not met. The chi-squared test was used to compare the percentage distributions of categorical data between the groups.

## Results

At the end of the study period, 294 patients had been diagnosed with brucellosis. Blood cultures were obtained from all patients, and *Brucella *spp. was detected in 40 patients (13.6%). Five patients had only blood culture growth and negative STA test results. There were 35 patients in Group 1, 254 patients in Group 2, and five patients in Group 3, respectively. As the number of patients detected in Group 3 was very low, these patients were not compared with the other groups. Table 1 shows the findings of the five patients in Group 3.

**Table 1:** Findings of the five patients in Group 3 CRP: C-reactive protein; AST: Aspartate aminotransferase; ALT: Alanine aminotransferase; WBC: White blood cells; PLT: Platelets.

	CRP mg/L (0-0.5)	AST(8-33) U/L	ALT (10-40) U/L	WBC (k/mcL) (4,000-10,000)	PLT (150,000-450,000) cell/mL	Fever	Sweating	Arthralgia	Localized involvement	Treatment	Treatment response
Patient 1	12,3	37	41	11,300	1,77,000	Positive	Positive	Positive	Negative	Rifampicin, doxycycline	Cure
Patient 2	11,3	18	17	4,900	1,44,000	Positive	Negative	Positive	Negative	Rifampicin, doxycycline	Cure
Patient 3	6,5	13	5	5,100	1,91,000	Positive	Negative	Positive	Negative	Rifampicin, doxycycline	Cure
Patient 4	5	526	436	4,300	1,15,000	Positive	Positive	Positive	Positive	Rifampicin, doxycycline	Cure
Patient 5	5	21	25	4,800	1,60,000	Positive	Positive	Positive	Negative	Rifampicin, doxycycline	Cure

Of the 289 patients evaluated, 128 (44.3%) were female and 161 (55.7%) were male, with an average age of 46 (18-82). While 233 (80.6%) of the patients described risks such as animal husbandry, fresh milk and cheese consumption, and occupational exposure, 61 (19.4%) patients did not have any risky contact. No statistically significant differences were observed in the demographic data between the groups.

When the presenting symptoms of the patients were analyzed, arthralgia was the most common clinical finding and was present in 68.6% of patients in Group 1 and 67.3% of patients in Group 2. This was followed by fever (65.7%-36.6%) and sweating (22.8%-20%), respectively. Only fever differed between the groups and was significantly higher in Group 1 (p = 0.001) (Table 2).

**Table 2:** Symptoms of the patients Group 1 and Group 2

	Group 1 (%)	Group 2 (%)	p-values
Arthralgia	68.6	67.3	p = 0.001
Fever	65.7	36.6	p > 0.01
Sweating	22.8	20	p > 0.01

The laboratory tests performed at the time of admission are shown in Table 3. C-reactive protein (CRP), aspartate aminotransferase (AST), and alanine aminotransferase (ALT) were significantly higher in Group 1, which had blood culture growth (p = 0.00, 0.023, p = 0.001).

**Table 3:** Difference in the laboratory findings between the two groups SD: Standard deviation; CRP: C-reactive protein; AST: Aspartate aminotransferase; ALT: Aspartate aminotransferase.

	Group 1 (n: 35), Mean ± SD (min-max)	Group 2 (n: 254), Mean ± SD (min-max)	p
CRP	4.47 ± 4.2 (0.1-17)	2.67 ± 3.91 (0-29)	0.000
AST	97.88 ± 227 (15-1,172)	33.3 ± 28.7 (9-219)	0.023
ALT	71.11 ± 151.6 (4-835)	35.29 ± 39 (2-236)	0.001
WBC	5,902 ± 2,473 (2,400-10,600)	7,747 ± 7,304 (290-78,000)	0.185
Platelets	216,500 ± 99,000 (46,000-473,000)	252,000 ± 86,300 (71,000-694,000)	0.121
Sedimentation	38 ± 32 (4-110)	29.8 ± 25.8 (1-127)	0.171

When examining the localized involvement sites in the patients, 79.6% had no localized involvement. Localized involvement was observed in 26% (n = 9/35) of Group 1 patients and 2% (n = 50/254) of Group 2 patients, with a significant difference between the two groups (p = 0.004). The other involved areas are listed in Table 4.

**Table 4:** Difference in localized regions of involvement between the groups

	Group		Total
	1	2	
No involvement	26	204	230
Arthritis	5	11	16
Orchitis	2	13	15
Endocarditis	1	0	1
Central nervous system	1	1	2
Spondylodiscitis	0	24	24
Retinitis	0	1	1
Total	35	254	289

Patients without localized involvement were administered a standard treatment regimen of rifampin (600 mg) and doxycycline (200 mg) for six weeks. We found that the treatment and duration varied according to the localized involvement sites. The treatment options for the patients in both groups are shown in Table 5.

**Table 5:** Treatment options in groups

		n
Group 1	Rifampicin + doxycycline	32
	Rifampicin + doxycycline + gentamicin	2
	Gentamicin + doxycycline	1
	Total	35
Group 2	Rifampicin + doxycycline	212
	Rifampicin + doxycycline + gentamicin	28
	Gentamicin + doxycycline	3
	Ceftriaxone	1
	Ciprofloksasin + rifampicin	1

Of the 46 patients, 26 patients were in the non-response group and 20 patients were in the relapse group. When the treatment responses were analyzed, 243 (84.1%) patients had complete recovery, while 46 (15.9%) experienced relapse or were non-responsive. Two of the 26 patients in the non-response group were in Group 1 and 24 were in Group 2. Two of the 20 patients in the relapse group were in Group 1 and 18 were in Group 2. When examining treatment responses by group, 88.5% of the patients in Group 1 and 83.4% in Group 2 had complete recovery, with no significant difference detected between the groups (p = 0.1). The treatment responses by the group are shown in Table 6.

**Table 6:** Treatment responses by groups

Treatment responses	Group		Total	p-value
	1	2		
Cure	31 (%88.5)	212 (%83.4)	243	0,1
Non-response	2 (%5.7)	24 (%9.4)	26	
Relapse	2 (%5.7)	18 (%7)	20	
Total	35	254	289	

## Discussion

The gold standard for diagnosing brucellosis is cultivating *Brucella *species in the blood or other tissue samples. In our study, only 40 (13.6%) of 294 patients had *Brucella *spp. growth in blood cultures. Growth rates in previous studies have been very variable, and our rate was found to be low compared to the literature [5]. The growth rate of *Brucella *species in blood or other tissue samples, which is the gold standard in the diagnosis of brucellosis, varies depending on the duration of the disease, the type and circulating amount of the causative agent, the length of the incubation period, and whether the patient has received antibiotic treatment before or not [6]. In the case of brucellosis, due to the long waiting time for bacterial production, low production rates, and laboratory constraints, growth in blood culture is not always possible. Therefore, serological methods have become the mainstay of diagnosis because of their high sensitivity and specificity, rapid results, simplicity, and low cost [7].

In our study, we found that although some clinical and laboratory findings differed in cases diagnosed with blood culture positivity compared to those diagnosed only by serology, it did not affect the treatment responses. Although our findings are not sufficient for a definitive judgment, our results suggest that serological diagnosis is not insufficient to make a correct diagnosis.

**Figure 1:**
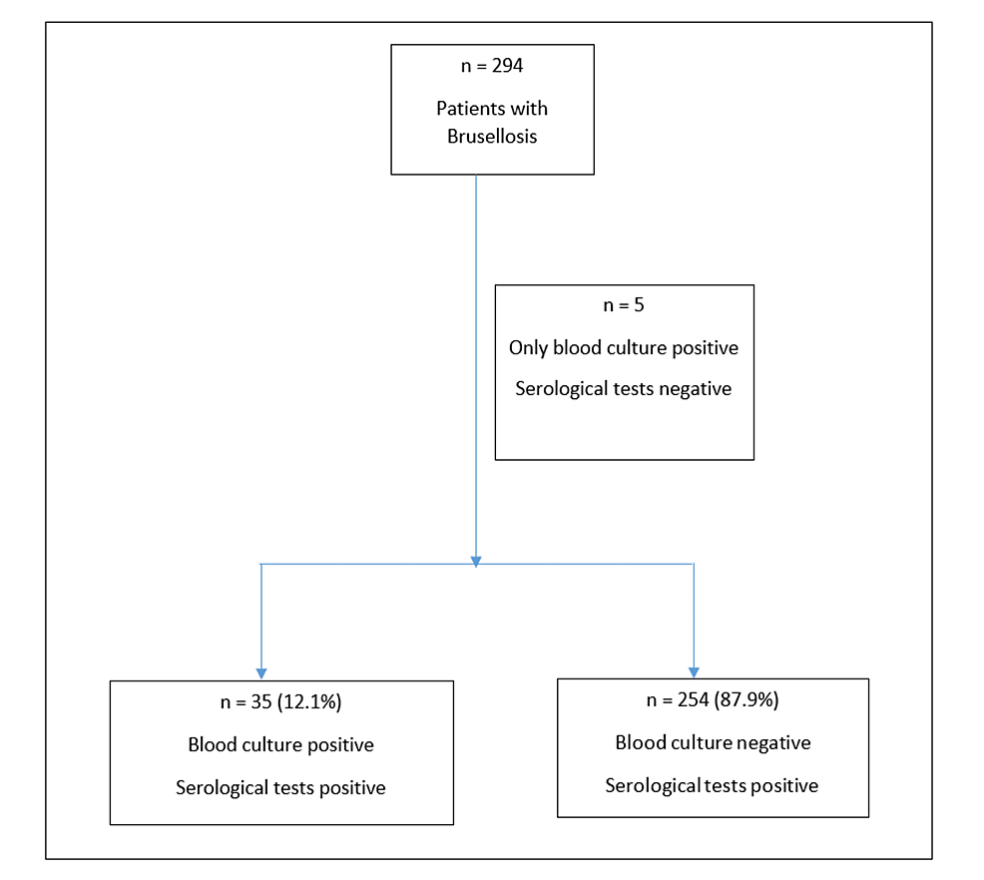
Patient distribution groups

Brucellosis is more common in young adults and has a similar male-to-female ratio. In our study, 128 (44.3%) patients were female and 161 (55.7%) were male, with an average age of 46 (18-82). Most of the patients (80.6%) had a history of risky exposure. Figure 1 shows the patient distribution groups.

Patients with brucellosis often present with fever, weakness, sweating, and joint pain. When the presenting symptoms of the patients were analyzed in our study, arthralgia, fever, and sweating were the most common clinical findings. Only fever differed between the groups (65.7%-36.6%) and was found to be significantly higher in Group 1, where blood culture growth was also present (p = 0.001). All five patients in Group 3, who were diagnosed with blood culture positivity only, had a fever. Similar to our study, in a study conducted by Özdem et al. with 189 pediatric patients diagnosed with brucellosis, cases with and without blood culture growth were compared in which fever was found to be significantly higher in patients with blood culture growth [8].

These data suggest that the rate of blood culture positivity is lower in patients without fever. Therefore, serological positivity may be considered sufficient for the diagnosis of patients without a fever.

Liver involvement is commonly observed in brucellosis patients. The clinical manifestations in the liver can range from mild transaminase elevation to severe hepatitis. Most of the brucellosis patients with liver involvement are asymptomatic, and a slight increase in transaminases may be detected [9]. In our study, AST and ALT showed a significant difference between the groups at the time of admission and were found to be significantly higher in Group 1, which had blood culture growth. Similarly, in a study of 195 patients by Sahinturk et al., transaminase levels were significantly higher in patients with blood culture growth [10]. When studies on brucellosis patients with positive blood cultures are analyzed, it is believed that systemic involvement is more common in patients with blood culture growth [11].

CRP is an acute phase reactant that increases under several acute and chronic inflammatory conditions. It has been reported to be elevated in many *Brucella *cases and is a marker for follow-up treatment [12]. In our study, CRP levels were significantly different between the groups, with significantly higher levels detected in Group 1. A significant relationship between CRP elevation and blood culture growth was found in a study of 150 patients with brucellosis [13]. Therefore, it should be kept in mind that brucellosis patients with elevated CRP, AST, and ALT in the laboratory may have a bacteremic course. In patients without significant CRP elevation, the diagnosis can be made based on serological positivity without waiting for growth in blood culture.

The diagnosis of *Brucella *is mostly based on clinical features of the disease and serological tests. However, the sensitivity of serological tests ranges from 65% to 95% and is associated with low specificity owing to the high antibody prevalence in the healthy population [14,15]. These problems complicate the interpretation of the serological test results. In support of this observation, although serological tests were negative in our study, *Brucella *spp. was grown in the blood cultures of five patients. However, in our study, the treatment response of patients diagnosed using clinical and serological tests in the culture-negative group was good. The majority of the patients (84.4%, n = 244) received standard treatment with rifampicin (600 mg) and doxycycline (200 mg). When the treatment responses were analyzed, 243 (84.1%) patients were cured, 46 (15.9%) patients were relapsed or unresponsive, and no significant difference was found between the groups in terms of treatment response. Based on these data, it can be concluded that it is appropriate to start treatment with a serological diagnosis without waiting for a positive blood culture for the diagnosis of brucellosis.

Limitations

The limitations of our study are that our study is retrospective, and the number of patients is small. The number of patients was not similar between the groups, which is associated with the retrospective nature of the study.

## Conclusions

In our study, among patients clinically diagnosed with acute/subacute brucellosis by serological methods, no significant difference was found in the treatment response and prognosis between patients with and without blood culture growth. Therefore, serological tests are reliable methods for diagnosing brucellosis in cases where blood culture is inconclusive. More extensive studies evaluating blood culture and serological methods are needed.
